# *Trans*-10, *cis* 12-Conjugated Linoleic Acid-Induced Milk Fat Depression Is Associated with Inhibition of PPAR**γ** Signaling and Inflammation in Murine Mammary Tissue

**DOI:** 10.1155/2013/890343

**Published:** 2013-05-14

**Authors:** Anil K. G. Kadegowda, M. Jawad Khan, Liliana S. Piperova, Beverly B. Teter, Sandra L. Rodriguez-Zas, Richard A. Erdman, Juan J. Loor

**Affiliations:** ^1^Department of Animal and Avian Sciences, University of Maryland, College Park, MD 20742, USA; ^2^Mammalian NutriPhysioGenomics, Department of Animal Sciences, Division of Nutritional Sciences, University of Illinois, Urbana, IL 61801, USA

## Abstract

Exogenous *trans*-10, *cis*-12-CLA (CLA) reduces lipid synthesis in murine adipose and mammary (MG) tissues. However, genomewide alterations in MG and liver (LIV) associated with dietary CLA during lactation remain unknown. We fed mice (*n* = 5/diet) control or control + *trans*-10, *cis*-12-CLA (37 mg/day) between d 6 and d 10 postpartum. The 35,302 annotated murine exonic evidence-based oligo (MEEBO) microarray and quantitative RT-PCR were used for transcript profiling. Milk fat concentration was 44% lower on d 10 versus d 6 due to CLA. The CLA diet resulted in differential expression of 1,496 genes. Bioinformatics analyses underscored that a major effect of CLA on MG encompassed alterations in cellular signaling pathways and phospholipid species biosynthesis. Dietary CLA induced genes related to ER stress (*Xbp1*), apoptosis (*Bcl2*), and inflammation (*Orm1*, *Saa2*, and *Cp*). It also induced marked inhibition of PPAR**γ** signaling, including downregulation of *Pparg* and *Srebf1* and several lipogenic target genes (*Scd*, *Fasn*, and *Gpam*). In LIV, CLA induced hepatic steatosis probably through perturbations in the mitochondrial functions and induction of ER stress. Overall, results from this study underscored the role of PPAR**γ** signaling on mammary lipogenic target regulation. The proinflammatory effect due to CLA could be related to inhibition of PPAR**γ** signaling.

## 1. Introduction

Dietary nutrients influence the quantity and composition of milk during lactation. Specifically, dietary lipids regulate milk lipid synthesis and milk fatty acid composition in different species of animals. Recently we showed that *trans* fatty acids (FA) and, in particular, *trans*-containing conjugated linoleic acid (CLA) isomers regulate murine mammary lipid metabolism to different extents [1]. CLA isomers are the positional and geometric isomers of linoleic acid, an 18-carbon FA with two double bonds. The conjugated double bonds in the CLA are responsible for their biological and biochemical activities. Of the different CLA isomers, the role of *trans*-10, *cis*-12-CLA in decreasing milk fat synthesis is well established.

The effects of dietary *trans*-10, *cis*-12-CLA on lipid metabolism in adipose and liver have been examined previously [2, 3]. Gene expression profiling studies in rodent adipose [2, 4], liver [5–7], and macrophages [8] have been conducted to help elucidate the molecular mechanisms elicited by *trans*-10, *cis*-12-CLA. In adipose and liver, *trans*-10, *cis*-12-CLA reduces adipogenesis, increases hepatic steatosis, and leads to insulin resistance, hyperinsulinemia, and inflammation [3, 9]. Studies on the effects of *trans*-10, *cis*-12-CLA in the mammary tissue of rodents and cows have largely focused on the changes pertaining to lipid metabolism. However, *in vitro* cell culture and *in vivo* studies have reported induction of mammary epithelial cell apoptosis at supraphysiological doses of CLA [10, 11].

We hypothesized that the effects of *trans*-10, *cis*-12-CLA in mammary tissue are not limited to lipid metabolism pathways and may involve other functional networks as has been observed in adipose tissue [4]. The specific objective of this study was to use microarrays and bioinformatics to characterize mammary and liver gene networks that are sensitive to supplemental *trans*-10, *cis*-12-CLA during lactation in mice.

## 2. Materials and Methods

The experimental procedures were approved by the Institutional Animal Care and Use Committee of the University of Maryland. The mammary and liver samples harvested from a previous experiment [1] were used in the present study. The details about the animals, diets, fatty acid composition of the diets, experimental design, and sample collection are described earlier [1]. Briefly, C57BL/6J mice fed the control diet from day 2 postpartum were randomly allotted to either control or a CLA-supplemented diet on day 6 postpartum. The control diet consisted of sucrose 590 g/kg, vitamin-free casein 200 g/kg, alphacel 50 g/kg, fat 100 g/kg (cocoa butter 14.3 g/kg, corn oil 30.9 g/kg, olive oil 34.8 g/kg, oleic acid 20 g/kg), AIN 76 mineral mixture 40 g/kg, AIN 76 vitamin mixture 15 g/kg, DL-methionine 3 g/kg, and choline bitartrate 2 g/kg. Oleic acid was replaced at 30% (wt/wt basis) by *trans*-10, *cis*-12-CLA in the CLA treatment. Oleic acid is incorporated into milk FA, but it was chosen because it has little effect on mammary lipid metabolism [12]. Litter size was adjusted to 6 or 7 pups to maintain uniform milk synthesis among mice. Lactating mice were fed the experimental diets from day 6 to day 10 postpartum. Milk samples were collected on day 6 and day 10 postpartum. On day 10 postpartum, the mice were sacrificed using isoflurane, and individual liver and mammary tissues were collected from dams, fast-frozen in liquid nitrogen, and stored at −80°C until RNA or lipid extraction.

### 2.1. RNA Extraction, Microarray, Quantitative Real Time Polymerase Chain Reaction (RT-qPCR), and Primer Design and Testing

The details of RNA extraction, microarrays, quantitative real time Reverse Transcription Polymerase Chain Reaction (RT-qPCR), and primer design and testing are presented in the Supplementary Material (see Supplementary Material available online at http://dx.doi.org/10.1155/2013/890343). Protocols for RNA extraction, RT-qPCR, and primer design and testing were as described previously [13]. Primer sequences for *Gpam*, *Insig1*, *Pparg*, *Scap*, *Scd*, and *Fasn* were published previously [1].  Table 1 contains information on additional target genes. The Mouse Exonic Evidence-Based Oligonucleotide (Oligator “MEEBO,” Mouse Genome Set, Illumina Inc.) platform containing 38,467 single-spotted oligonucleotides targeting 35,302 genes was used for transcript profiling. Methods for aminoallyl labeling of cDNA, microarray hybridizations, and scanning were as described earlier [14].

### 2.2. Statistical Analysis

**Figure 1:**
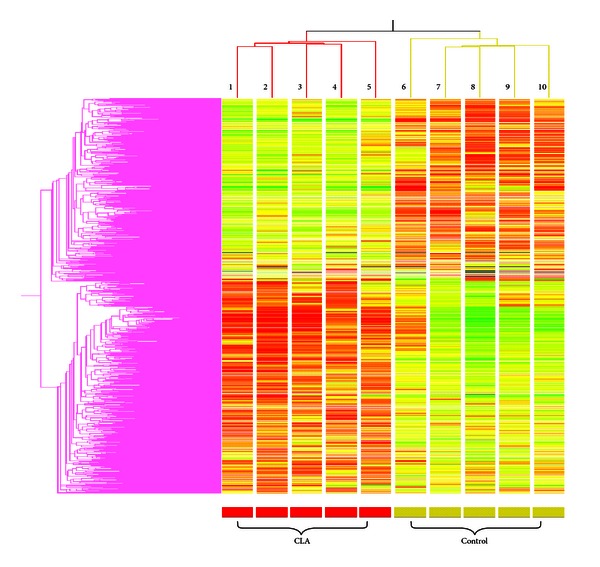
Hierarchical clustering analysis of DEG (1,496) in mammary tissue from individual lactating mice fed *trans*-10, *cis*-12-CLA (CLA) or the control diet (CTR). The colors of the heat map represent the expression of each DEG in mammary tissue versus reference, with red, green, and the various hues denoting greater (red) to lower (green) relative expression (tissue/reference). The average Pearson correlation across animals in the CLA group was 0.38 and for those in the CTR 0.41. The numbers represent an individual mouse within the CLA or CTR group. The clustering by gene is denoted by the pink tree on the left of the figure.

**Figure 2:**
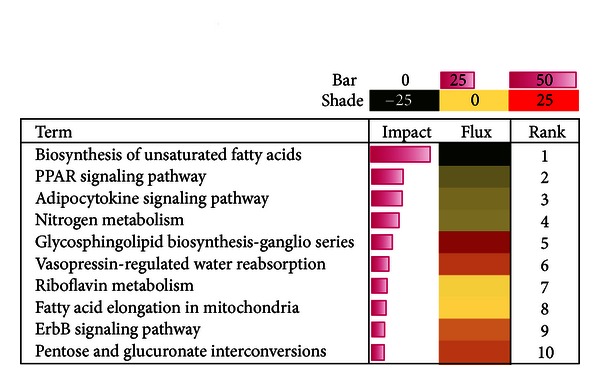
Top ten affected KEGG pathways/terms by *trans*-10, *cis*-12-CLA in mammary tissue. The horizontal bars denote the impact of DEG on the KEGG pathways. The larger the horizontal bar, the greater the impact. The direction of the impact is indicated under flux, green = inhibition and red = activation. The intensity of the color indicates the extent of inhibition (if green) or activation (if red); that is, darker the color, the greater the effect on the pathway.

**Figure 3:**
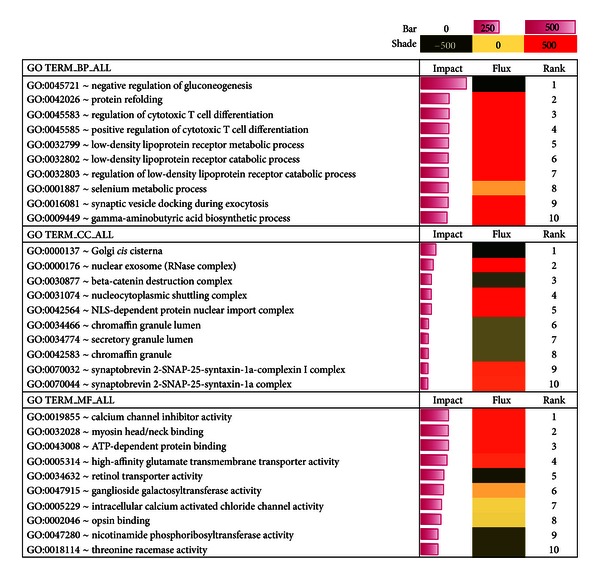
Top ten affected GO terms (DAVID) by *trans*-10, *cis*-12-CLA in mammary tissue. The horizontal bars denote the impact of DEG on the GO terms. The larger the horizontal bar, the greater the impact. The direction of the impact is indicated under flux, green = inhibition and red = activation. The intensity of the color indicates the extent of inhibition (if green) or activation (if red); that is, darker the color, the greater the effect on the GO terms. BP: biological processes; CC: cellular components; MF: molecular functions.

Data from 40 microarrays (two dyes, two tissue samples/mouse, for a total of 10 animals) used for analysis were initially normalized for dye effects using the median of control elements on the microarray. Subsequently, the log2 normalized ratios of mammary versus reference (i.e., RNA mixture of different tissues including mammary) signal intensities were adjusted for global dye and microarray effects and normalized by Lowess. The data were analyzed using MIXED procedure (SAS Institute Inc., Cary, NC, USA). The fixed effects of the model included tissue and dye while the random effects were mouse and microarray. Raw *P* values for the tissue effect were adjusted using Benjamini and Hochberg's False Discovery Rate (FDR). Differences in relative expression between CLA and control were considered significant at an FDR-adjusted *P* ≤ 0.20. For a more stringent characterization of differentially expressed genes (DEGs), a ≥1.5-fold difference in mRNA expression was set as threshold. We used a more liberal cutoff for the liver data set as very few genes met the criterion used for the mammary tissue. The liver data were assessed using a *P* < 0.05 without an FDR adjustment but with a threshold of ≥1.4-fold difference to determine affected genes. Visualization of within-group variance for DEG in mammary gland tissue and for the entire set of transcripts in liver tissue was performed via hierarchical clustering and box-plot analysis using GeneSpring GX (Agilent Technologies; Figures 1–3). Data from qPCR after normalization with internal control genes (using the geometric mean of *Stx8*, *Plod3*, and *Ngb*) were analyzed using MIXED procedure (SAS Institute Inc., Cary, NC, USA). The complete dataset files have been deposited at the MIAME database (http://www.ebi.ac.uk/arrayexpress/; username: reviewer_E-MEXP-3622, password: veitxmnm).

### 2.3. Bioinformatics Data Mining

Bioinformatics was conducted using the Dynamic Impact Approach (DIA) according to Bionaz et al. [15]. Briefly, this analysis relied on the publicly available resources Kyoto Encyclopedia of Genes and Genomes (KEGG) database and the Database for Annotation, Visualization and Integrated Discovery (DAVID v6.7, 20). Entrez gene IDs were used to identify individual sequences. The KEGG resource has pathway information associated with each gene ID in *Mus musculus*. There are six major pathway categories, namely, metabolism, genetic information processing, environmental information processing, cellular processes, organismal systems, and human diseases. In addition, we used Ingenuity Pathways Analysis (IPA) relying on the entire microarray data set with associated statistical *P* values. The DAVID resource provides typical batch annotation and gene-GO term enrichment analysis to highlight the most relevant GO terms associated with a given gene list. The latest version provides extended annotation content coverage including GO terms, protein-protein interactions, protein functional domains, disease associations, biological pathways, sequence general features, homologies, gene functional summaries, and tissue gene expression.

The ToppGene suite (http://toppgene.cchmc.org/) was used for functional enrichment analysis of data from liver tissue at an uncorrected *P* < 0.05 and a threshold of ≥1.4-fold in CLA versus control. ToppGene suite is a web-based portal that provides 17 categories of annotations, including GO-molecular function, GO-biological process, GO-cellular component, human phenotype, mouse phenotype, protein domains, pathways, pubMed cocitations, protein-protein interactions, cytoband, transcription factor binding sites, gene family coexpression, computational expression correlations, micro-RNA targets, drug, and disease. The database details for each category of annotations are presented in the ToppGene web portal (http://toppgene.cchmc.org/).

## 3. Results

We have previously reported the effect of *trans*-10, *cis*-12-CLA on milk production, milk fat content, milk fatty acid composition, liver weight, and liver FA composition [16]. Briefly, compared with the control, CLA decreased milk fat percentage by 44% and reduced the proportion of *de novo* synthesized FA (FA ≤ C 16:0) while increasing 20:4n-6 (Table 2). The *trans*-10, *cis*-12-CLA was preferentially taken up by the mammary tissue (1.42 g/100 FAME in milk) compared with liver (0.20 g/100 FAME). However, the liver weight increased by 32% in mice fed CLA as a consequence of increased lipid content (0.47 g FAME/g liver, dry) compared with control (0.24 g FAME/g liver, dry). Furthermore, the proportions of FA 18:0, 18:3, and 20:4n-6 decreased (*P* < 0.01) in the liver of mice fed CLA diet (Table 2).

### 3.1. Mammary Tissue

In the mammary tissue, IPA identified 27,566 of 35,302 sequences on the MEEBO platform. The CLA diet resulted in a differential expression of 1,496 genes (FDR = 0.20, *P* < 0.01) of which 143 genes differed from control more than 1.5-fold (data not shown). Hierarchical clustering of the 1,496 DEG (Figure 1) revealed a high-degree of consistency among animals in the response to CLA or control. This analysis also allowed visualization of two obvious groups of genes that were downregulated or upregulated by CLA compared with controls. The IPA analysis (Table 3) revealed that *trans*-10, *cis*-12-CLA caused a marked effect in mammary tissue on genes associated with acute-phase response signaling, regulation of actin-based motility by rho, glycosphingolipid biosynthesis, RAR, and LXR/RXR activation.

The top upregulated genes were defensin beta (*Defb*), hypoxanthine phosphoribosyl transferase 1 (*Hprt1*), orosomucoid 1 (*Orm1*), sphingosine kinase, kinesin family member 26b (*Sphk1*), ceruloplasmin (ferroxidase) (*Cp*), and serum amyloid A2 (*Saa2*) (Table 4). The top down-regulated genes were Major urinary protein 4 (*Mup4*), Major urinary protein 2 (*Mup2*), Carbonic anhydrase III (*Ca3*), Adiponectin (*Adipoq*), TATA box binding protein (*Tbp*), Stearoyl-CoA desaturase 1 (*Scd1*), ELOVL family member 5, elongation of long chain fatty acids (*Elvol5*), and tubulin (*Tubg2*) (Table 4).

#### 3.1.1. Bioinformatics

A total of 217 pathways in the KEGG database (murine genome) contained 30% or more genes represented on the microarray platform; these were deemed appropriately to be covered for further analysis. Among the most-impacted pathways (average impact 5.78 ± 2 SD (16.4)) that were affected due to feeding CLA versus control were biosynthesis of unsaturated fatty acids, PPAR signaling pathway, adipocytokine signaling pathway, and nitrogen metabolism (Figure 2). Overall, those pathways were inhibited due to dietary CLA. Pathways are considered moderately affected by feeding CLA (5.78 ± 1 SD (11.1)) and with clear activation included glycosphingolipid biosynthesis and vasopressin-regulated water reabsorption. Other pathways are considered modestly affected by feeding CLA (5.78 ± 0.5 SD (8.4)) including riboflavin metabolism, fatty acid elongation in mitochondria, ErbB signaling, and pentose and glucuronate interconversions, which were activated (Figure 2).

The DAVID categories biological process (GOTERM_BP_ALL), molecular function (GOTERM_MF_ALL), and cellular component (GOTERM_CC_ALL) are shown in Figure 3. Within biological processes are “biological goals” such as regulation of gluconeogenesis which is accomplished by ordered assemblies of molecular functions. Molecular Function describes the tasks performed by individual gene products, for example, calcium channel inhibitory activity. The cellular component classification type involves subcellular structures, localization, and macromolecular complexes, for example, Golgi cisterna.

The top 10 GO terms from our analysis are shown in Figure 3, and the approach for calculating impact and flux within each GO category has been described previously [14, 15]. Briefly, the length of the impact bar denotes the relative biological significance of the gene identifiers in each category; that is, a bigger bar reflects a more important biological term based on DEG. Similarly, the relative direction of the flux (activation or inhibition) is denoted by the intensity of color as reported in Bionaz et al. [15]. Results using DIA indicated that, within biological processes, only 2 of the top 10 most-impacted categories were inhibited, that is, negative regulation of gluconeogenesis and selenium metabolic process, whereas protein refolding and regulation of cytotoxic T cell differentiation were highly impacted and activated (Figure 3). Interestingly, the GO terms within cellular component were the least impacted among the three GO categories and mostly seemed to be inhibited in response to feeding CLA, for example, golgi *cis* cisterna and *beta*-catenin destruction complex (Figure 3). Within molecular functions, feeding CLA led to an activation of calcium channel inhibitor activity, myosin head/neck binding, and ATP dependent protein binding, whereas it inhibited retinol transporter activity and nicotinamide phosphoribosyltransferase activity.

#### 3.1.2. Microarray Verification

The microarray results were verified by quantitative real time RT-PCR assays for genes regulating lipid metabolism, apoptosis, inflammation, acute-phase response signaling, and transcription regulation (Table 5). Of the 21 genes tested, 17 genes were similar to those of microarray and four were false-positives (Table 5).

### 3.2. Liver

The effect of *trans*-10, *cis*-12-CLA on the liver transcriptome was modest at the same criterion used for selecting DEG of mammary tissue. Hierarchical clustering (not shown) across individual animals and the box-plot analysis (not shown) revealed a high-degree of animal variation in response to CLA or control, which is not unexpected based on previous studies with lactating mice [17]. We were unable to cluster by gene due to the large size of the dataset (38,185 elements); that is, there were no statistically significant genes in liver at the stringent cutoff used with mammary data. There were 170 DEGs when the selection criteria were relaxed (uncorrected *P* < 0.009, 1.5-fold difference in CLA versus control) with 72 upregulated and 98 down-regulated. The CLA diet did not affect genes related to FA synthesis (e.g., *Fasn*, *Acc*, and *Scd1*; data not shown) even though there was increase lipid accumulation in the liver. Some of the genes related to FA oxidation *(Acox1*, *Cpt1c,* and *Fmo3*) were down-regulated (data not shown). Among the genes related to FA transport, CD36, *Fabp1,* and *Fabp2* were upregulated while *Fabp4* was down-regulated (data not shown).

Feeding CLA increased liver mass and altered hepatic FA composition (Table 2). The greater liver mass was due in part to lipid accumulation and was associated with several alterations in gene expression. For instance, the “mouse phenotype” feature of the ToppGene suite used for the gene enrichment analysis identified hepatic steatosis, abnormal Kupffer cell morphology, abnormal liver sinusoid morphology, abnormal mononuclear phagocyte, abnormal circulating creatinine level, and abnormal circulating amino acid level as the top terms affected by CLA feeding (Table 6). The most impacted pathways by CLA were electron transport chain, glucose regulation of insulin, glyoxylate cycle, biosynthesis of unsaturated FA, alanine and aspartate metabolic pathway, erythrocytes differentiation pathway, and tyrosine degradation (Table 7). The finding of an overall upregulation of the unsaturated FA biosynthesis pathway was due to the fact that the pathway includes several upregulated genes (e.g., *Acot2*, *Fads1*, *Fads2*, *Agpat2,* and *Elovl5*; data not shown) as identified by ToppGene.

The GO categories GO: molecular function, GO: biological process, and GO: cellular component are presented in Table 8. Feeding CLA activated molecular functions related to oxidoreductase activity, chaperone binding, unfolded protein binding, pyrophosphatase activity, but inhibited cofactor binding, pyridoxal phosphate binding, and vitamin B6 binding. CLA feeding upregulated the biological processes related to oxoacid-, carboxylic acid-, organic acid-, and ketone-metabolic processes in addition to cellular catabolic process. Mitochondrion was identified as the most-impacted cellular component in the liver of mice fed CLA.

## 4. Discussion

We observed contrasting effects of *trans*-10, *cis*-12-CLA on liver and mammary tissue, two predominantly lipid-synthesizing tissues in lactating mice. In the mammary tissue, the reduction in milk lipid content was characterized by lower concentrations of *de novo* synthesized fatty acids (FA ≤ C 16:0) as a consequence of inhibition of several genes related to lipid synthesis (data not shown). Lipid metabolism was the main cellular function affected by* trans*-10, *cis*-12-CLA, and the large impact and inhibition of biosynthesis of unsaturated fatty acids KEGG pathway confirmed such effect. The bioinformatics analyses underscored that a major effect of *trans*-10, *cis*-12-CLA on mammary tissue encompassed alterations in signaling pathways and phospholipid species biosynthesis.

Substantial regulation of milk lipid synthesis in lactating mice occurs at the mRNA level, and there is evidence suggesting that part of the pathway is regulated by SREBP1 [18, 19]. The lower expression of *Srebp1* and its downstream genes in previous studies provided evidence of its role in murine milk lipid synthesis. However, the effect of *trans*-10, *cis*-12-CLA on SREBP1 is indirect as FA cannot bind to this transcription factor [20]. In contrast, FA are natural ligands of the nuclear receptor PPAR*γ* which is known to regulate lipogenesis in some cell types [21–23]. The overall inhibition of PPAR signaling KEGG pathway, the reduction in *Ppar*γ**expression and its downstream genes regulating FA uptake (*Fabp4*), FA synthesis (*Fasn*), FA desaturation (*Scd*), and lipid droplet formation (*Cav1*) due to *trans*-10, *cis*-12-CLA, all support a role for PPAR*γ* in murine mammary lipid metabolism.

We have previously shown that activation of PPAR*γ* could upregulate mammary lipogenic gene networks in bovine mammary epithelial cells [13]. CLA has also been shown to act as a PPAR*γ* activator in some cell types [24]. However, an important consideration in the context of PPAR*γ* regulation of murine mammary lipid synthesis is the fact that the mammary gland of this species contains a substantial amount of adipocytes which may in fact be an important source of PPAR*γ* and also adipocytokine mRNA expression [25].

Glycosphingolipid biosynthesis-ganglio series was the most activated KEGG pathway in addition to being one of the most-impacted pathways by *trans*-10, *cis*-12-CLA. Glycosphingolipids are key components of the apical plasma membrane and subcellular components including vesicles [26]. Glycosphingolipids are involved in molecular functions such as differentiation, apoptosis, and cell-cell interaction [26]. In murine mammary tissue, ganglioside GD1*α* is specifically increased during lactation and is highly concentrated in the milk fat globule membranes and helps prevent aggregation of milk fat globules [27]. We have previously observed a shift in the lipid globule distribution towards an increase in the number of smaller lipid droplets in the secreted milk as well as intracellular mammary epithelium in mice fed *trans*-10, *cis*-12-CLA [17], and this could be due to altered glycosphingolipid biosynthesis in addition to reduced lipogenesis.

Based on microarray data from murine mammary tissue Rudolph et al. [19] suggested that gluconeogenesis per se is not an important function in murine mammary epithelium. However, the enzyme PCK1 which is a key component of this pathway in liver also is central for the generation of glycerol-phosphate for esterification during the process of glyceroneogenesis [28]. Thus, it is not surprising that the top most-impacted GO biological process due to dietary CLA was inhibition of negative regulation of gluconeogenesis; that is, supply of glucose to mammary gland might have been decreased with a consequent increase in use of other carbon sources (e.g., lactate) to generate glycerol-phosphate.

In addition to its effects on lipid metabolism, in the IPA analysis* trans*-10, *cis*-12-CLA affected cellular processes related to cell cycle progression, cell-cell interactions, and cell assembly and organization (data not shown). These effects of CLA were probably through signaling pathways regulating ErbB receptors and the actin cytoskeleton (Figure 2). ErbB signaling was identified as one of the top 10 impacted KEGG pathways in our study. The ErbB signaling pathway in murine mammary gland is involved in the control of cell survival, cell-cell interaction and cellular differentiation, cell cycle progression, and morphogenesis [29, 30]. Activation of the ErbB pathway is considered beneficial for cellular proliferation in damaged tissue [30]; thus, it could be possible that the activation of this pathway in our study was a compensatory response due to CLA. Proliferation of mammary epithelium and ductal hyperplasia has also been observed in CLA-fed mice independent of alterations of ErbB expression [31]. Ovariectomized mice fed CLA experienced an estrogen-independent allosteric mammary growth characterized by ductal elongation due to enlarged proliferative terminal end buds [32]. In addition, CLA feeding was associated with increased incidence of tumor development and progression [31, 33].

Regulation of actin-based motility by rho was one of the top 5 canonical pathways identified by IPA analysis. Dietary CLA upregulated expression of actin B (*Actb)* and actin G (*Actg)*, which play an important role in formation of stress fibers connecting extracellular matrix to the intracellular medium. Profilin (*Pfn*) is involved in actin filament polymerization [34]. Rho proteins regulate *Actb*, *Actg,* and *Pfn* [35] and can affect actin polymerization which can actively drive vesicle movement in cells [36]. The ras homolog D (*RhoD*), which is involved in endosomal dynamics and rearrangement of actin cytoskeleton, was also upregulated by CLA in this study. RhoD is involved in the regulation of both membrane traffic and cytoskeleton in the cell [37]. Overexpression of RhoD induced remodeling of actin cytoskeleton accompanied by increased endosomal fission and scattering of vesicles in the cells [37]. Thus, our results suggested that CLA either directly or indirectly caused a substantial remodeling of cellular structures. It could be possible that such effects were partly due to changes in unsaturated FA availability, for example, for cellular membrane formation.

**Figure 4:**
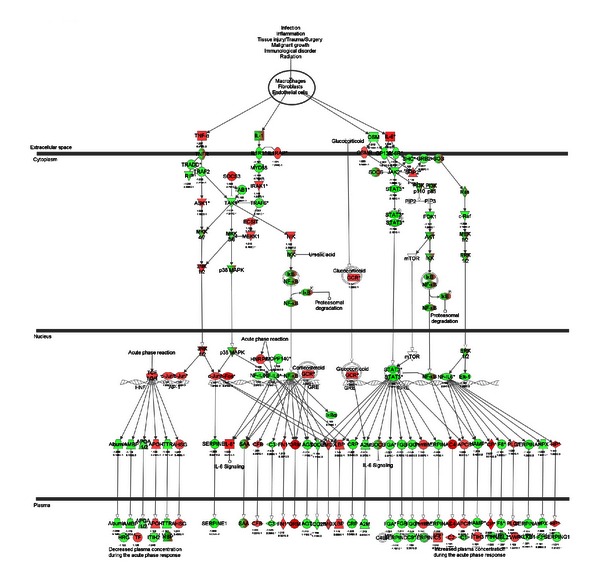
Acute-phase response signaling pathway (IPA) expression in the mammary tissue of lactating mice fed *trans*-10, *cis*-12-CLA. Green indicates downregulation while red indicates upregulation.

*Trans*-10, *cis*-12-CLA causes marked dilation of endoplasmic reticulum (ER) in the mammary epithelial cells and upregulates x-box binding protein 1 (Xbp1) expression. In addition, CLA is also known to induce the ER stress response by increasing splicing of XBP1 mRNA and activation of c-Jun N-terminal kinase (JNK) signaling [38, 39]. In our study, a greater expression of *Xbp1* and *Jnk1*, genes coding key proteins in the ER stress response, due to CLA suggested the induction of an unfolded protein response (UPR) in the ER. That idea is supported by the downregulation of calnexin (*Canx)*, a molecular chaperone which mediates the proper folding of nascent proteins in ER, coupled with upregulation of *BiP* and *HSP90AA1* which bind to misfolded proteins. Thus, the activation of UPR with CLA might have been associated with greater ER stress and underscored a marked derangement in posttranslational modification of proteins. A key feature of the UPR is cellular inflammation [40], and the activation of cytotoxic T cell differentiation along with several acute-phase proteins in our study seems to confirm that CLA exerted a proinflammatory state in mammary cells (Figure 4). In this context, the inhibition of golgi cis cisterna, beta-catenin destruction complex, and nuclear exosome could have been a response to ER stress and inflammation.

*Trans*-10, *cis*-12-CLA inhibits cellular proliferation and induces apoptosis in primary mammary epithelial cells [10]. Different mechanisms have been proposed for the induction of apoptosis by CLA: (a) p53-dependent [41, 42]; (b) mitochondrial pathway targeting Bcl2 [43]; and (c) ER response involving induction of Xbp1, phosphorylation of eIF2*α*, induction of CHOP, and the cleavage of caspase 12 [38]. In this study, in addition to the induction of ER stress response, a gene related with the mitochondrial pathway, for example, *Bcl2*, was also affected. The Bcl protein decreases the activation of caspase 3 involved in apoptosis [44], and a mouse *Bcl2*-mutant strain has greater rates of apoptosis in cells [45].

The acute-phase response signaling was the major canonical pathway affected by *trans*-10, *cis*-12-CLA in the mammary tissue (Figure 4). The top three upregulated genes *Orm1* (3.7-fold), *Cp* (2.9-fold), *Saa2* (2.6-fold), in addition to *C4b *(2.1-fold), *Orm2* (2.27-fold), and *Saa1* (2.3-fold) are classified as acute-phase proteins (Figure 3). Some of the top down-regulated genes *Mup4 *(−8.4-fold)*, Mup2 *(−4.6-fold), and *Rbp4 *(−1.8) are well-established negative regulators of acute-phase proteins [40, 46] (Figure 3). Acute-phase proteins are induced in response to tissue injury leading to robust inflammatory responses [40, 47]. In addition to acute-phase proteins *Defb *(4.6-fold), involved in antimicrobial immune response [48], increases during chronic wounds to promote healing [49, 50], and *Sphk1* (3.6-fold) enhances proinflammatory cytokines [51].

The murine mammary tissue is characterized by an upregulation of acute-phase proteins during the initial phase of involution, but the exact role of such response has not been determined [52–54]. In our study, a proinflammatory effect induced by CLA on mammary tissue could have led to decreased milk synthesis capacity of the epithelial cells and a subsequent effect on the growth of pups nursing mice fed *trans*-10, *cis*-12-CLA [1]. The marked accumulation of 20:4n-6 in milk of CLA-fed mice might have played a role in the proinflammatory response observed, for example, by enhancing the production of oxidized lipid products [55]. The accumulation of 20:4n-6, however, contrasts with the marked decrease in its concentration in milk of lactating dairy cows receiving an exogenous infusion of CLA that reduced milk fat synthesis [56].

The acute-phase response proteins are closely regulated at the transcriptional level by STAT3 and NFkB [57]. ER stress can induce upregulation of acute-phase proteins through NFkB activation [58]. None of these transcriptional regulators was upregulated with CLA. However, there was upregulation of the proinflammatory cytokines *IL6 *and *TNF*. IL6 is known to exert its action through increasing STAT3 [52]. During the acute-phase response, TNF decreases the expression of nuclear receptors PPAR*γ*, PPAR*α*, RXR*α*, and LXR*α* [43] which in turn could affect the lipid-synthesizing transcriptome. In addition to its effects on lipid metabolism, PPAR*γ* dampens inflammation via transrepression of proinflammatory molecules [21]. For instance, PPAR*γ* inhibits inflammation by direct interaction with AP1 and NFkB, thus, preventing their binding to response elements on inflammatory genes [21]. Activation of PPAR*γ* by synthetic PPAR*γ* agonists reduces the expression of inflammatory cytokines and chemokines in bovine mammary epithelial cells [59]. While FA are considered as natural ligands of PPAR*γ* [21–23], the lower PPAR*γ* expression and greater acute-phase protein expression, which confirmed the existence of a marked degree of inflammation, suggested that *trans*-10, *cis*-12-CLA antagonizes PPAR*γ*.

Further work is needed to determine if the ability of PPAR*γ* to repress proinflammatory molecules is diminished due to its interaction (e.g., competitive inhibition) with *trans*-10, *cis*-12-CLA. Overt inflammation might reduce transcription of anti-inflammatory nuclear receptors (e.g., feedback inhibition) leading to negative effects on cellular mechanisms including lipid synthesis and secretion. In lactating mouse mammary tissue targeted deletion of *Ppar*γ** resulted in greater concentration of proinflammatory lipids in milk leading to reduced pup growth and induction of inflammation. Those results highlighted the importance of PPAR*γ* signalling during lactation [55]. Some of the effects of *trans*-10, *cis*-12-CLA in our study might have been regulated through its effects on PPAR*γ*.

The effect of *trans*-10, *cis*-12-CLA on liver was dramatic as CLA caused accumulation of lipid in the span of four days. The associated increase in liver mass due to TAG accumulation suggested induction of hepatic steatosis in CLA-fed animals. This was confirmed with the gene functional analysis as “hepatic steatosis” was one of the top terms associated with the “mouse phenotype” category. Primary biliary cirrhosis and liver cirrhosis were the top terms in the “disease” category suggesting that *trans*-10, *cis*-12-CLA feeding predisposed the mice to these conditions. The onset of hepatic steatosis could predispose liver to inflammation and oxidative stress leading to steatohepatitis and eventually to liver cirrhosis [60, 61]. The development of hepatic steatosis is specific for *trans*-10, *cis*-12-CLA feeding but not to other CLA isomers [62]. Hepatic steatosis has been previously reported in growing mice after 14 days of *trans*-10, *cis*-12-CLA feeding [63].

Potential mechanisms involved in the onset of hepatic steatosis due to *trans*-10, *cis*-12-CLA have been reviewed recently [3], but it is important to recognize that all those previous studies have been conducted with nonlactating animals. The reduction in FA oxidation coupled with increased uptake of FA may be one of the reasons for hepatic lipid accumulation [3]. Decreased FA oxidation could lead to accumulation of intracellular diacylglycerol concentration which is implicated in hepatic insulin resistance through activation of protein kinase C [26]. In a recent study, CLA-induced hepatic steatosis was associated with an increase in diacylglycerol content and membrane associated protein kinase C [64] suggesting that CLA feeding could potentially lead to hepatic insulin resistance.

In this study, mitochondria, the primary organelle involved in FA oxidation, were the most-impacted cellular component followed by ER and peroxisomes. Mitochondrial dysfunction causes hepatic steatosis and has been implicated in the progression from nonalcoholic fatty liver disease (NAFLD) to non-alcoholic steatohepatitis (NASH) [65]. The top affected pathways (e.g., electron transport chain and glucose activation of insulin secretion), molecular functions (e.g., oxidoreductase activity), biological processes (e.g., oxoacid metabolic process, and cellular catabolic process, cellular ketone metabolic process), and diseases (e.g., Leigh disease, Leigh syndrome) are related to mitochondria further confirming that *trans*-10, *cis*-12-CLA feeding affected hepatic mitochondrial functions.

The ER was the second most affected cellular organelle in the liver of *trans*-10, *cis*-12-CLA-fed mice. The activation of molecular functions such as chaperone binding and unfolded protein binding suggests induction of ER stress response in hepatic tissue. Induction of ER stress could further deteriorate the steatotic condition by augmenting hepatic lipogenesis via activating proteolytic cleavage of SREBP1 [66], decreasing hepatic secretion of TAG by reducing apoB secretion [67] and upregulation of Xbp1 [68]. The transcriptional regulator Xbp1, a known regulator of the unfolded protein response, regulates the expression of lipogenic genes *Scd1*, acetyl*-*CoA carboxylase beta (*Acc2*), and diacylglycerol acetyltransferase 2 (*Dgat2*), independent of SREBP1 and ChREBP [68]. Also, a shift in hepatic FA composition towards saturated FA disrupts ER homeostasis and promotes ER stress [69, 70]. An increased ratio of saturated to unsaturated FA is considered as a secondary hit in the progression of steatosis to steatohepatitis [69, 71]. *Trans*-10, *cis*-12-CLA-fed mice in our study had greater hepatic palmitate concentration (67% over control) thereby increasing the ratio of saturated FA. Thus, those data confirm previous reports that the induction of ER stress could be caused in part by altered hepatic FA profile.

As indicated above, the presence of adipocytes in the mammary tissue used for transcriptomics is an important consideration when interpreting our data, particularly as it relates to PPAR and adipocytokine signaling. Adipose tissue constitutes ca. 20% of the lactating murine mammary tissue in early lactation [69] and in rodents is indispensable for the normal function of the mammary epithelium [16, 72]. It has been suggested that the gene expression changes in a complex tissue like rodent mammary could be a function of changes in the gene expression within a particular cell type (i.e., mammary or adipose) or the proportion of the given cell type (i.e., mammary or adipose) [25]. This could be an important factor during different stages of mammary function and development when the abundance of the two cell types varies substantially [25]. Our study was conducted during peak lactation when the adipose content is at its lowest, but further studies are needed to delineate more closely the impact of *trans*-10, *cis*-12-CLA on the relative abundance of mammary and adipose cell types in the rodent mammary gland during peak lactation.

## 5. Conclusions

During lactation the effect of CLA on the liver was similar to those observed previously in growing mice. The hepatic gene enrichment functional analysis revealed induction of hepatic steatosis, perturbations in the mitochondrial functions, and induction of ER stress. The major effect of CLA on mammary tissue encompassed alterations in cellular signaling pathways, phospholipid species biosynthesis, and a marked degree of inflammation and ER stress. Such responses might be associated with enhanced cellular apoptosis and precocious involution of the mammary gland. Inhibition of PPAR*γ* signaling was associated with decreased milk fat synthesis and uncontrolled inflammation.

## Supplementary Material

Supplementary Table 1: Top molecular and cellular functions (FDR=0.2, P<0.001 and fold change >1.5) up-regulated in the mammary tissue of lactating mice fed trans-10, cis-12-CLA.
Supplementary Table 2: List of genes with stable gene expression across all the samples.
Supplementary Table 3: Gene list for selecting internal controls without any interaction by IPA analysis.

## Figures and Tables

**Table 1:** Accession number, gene symbol, and primer sequences of target genes. Underlined are exon-exon junctions.

Accession number	Gene	Hybridization	Sequence (5′ to 3′)	Size (bp)
NM_009735	*B2m *	F.109	GCTATCCAGAAAACCCCTCAAA	100
		R.208	GCGGGTGGAACTGTGTTACG	
NM_007527	*Bax *	F.346	GAGCTGCAGAGGATGATTGCT	100
		R.445	CCCAGTTGAAGTTGCCATCAG	
NM_009780	*C4b *	F.4023	ACTGAGGAGAAAGCACTGAACGT	127
		R.4149	TGCCCAGGGAGAACTTTAGC	
NM_001042611	*Cp *	F.1173	CTGTTCCCTGCCACCCTAATT	101
		R.1273	TGCAACCCAGCTTTCAGATG	
NM_007843.2	*Defb1 *	F.102	AAACTCATTACTTTCTCCTGGTGATGA	100
		R.201	TATTGATCTGTTCTTCGTCCAAGACT	
NM_021284	*Kras *	F.176	GGAGAGAGGCCTGCTGAAAA	100
		R.275	GTGATTCTGAATTAGCTGTATCGTCAA	
NM_001045550	*Mup2 *	F.480	CTCTATGGCCGAGAACCAGATT	120
		R.599	GAGGCAGCGATTGGCATT	
NM_008768	*Orm1 *	F.357	AAGTATGAAGGAGGAGTAGAAACCTTTG	100
		R.456	CCCGTTTCTTCTCATCCTTGAG	
NM_011016	*Orm2 *	F.501	GAGCTGCGGGAAGTATTCCA	93
		R.593	ACTGCACCTGTCCTTTTTCCA	
NM_009045	*Rela *	F.1191	GCCCATGGAGTTCCAGTACTTG	124
		R.1314	TTCAGTTGGTCCATTGAAAGGA	
NM_009117.3	*Saa1 *	F.19	CCAGGAGACACCA**GG**ATGAAG	100
		R.118	AAGCCTCGTGAACAAATGAAAAA	
NM_011314.1	*Saa2 *	F.21	AGGAGACACCA**GC**AGGATGAA	100
		R.120	AGCCTCCCCAATAAATGAAAAAA	
NM_011489	*Stat5b *	F.426	ACCATGTCTGTGACCCAAAGTAAA	100
		R.525	CACAACCTACAGAGCCCGAATC	
NM_013693	*Tnf *	F.334	AGGGATGAGAAGTTCCCAAATG	101
		R.434	GCTACAGGCTTGTCACTCGAATT	
NM_011480.2	*Srebf1 *	F.2066	CATGCCATG**GG**CAAGTACAC	105
		R.2170	TGTTGCCATGGAGATAGCATCT	

**Table 2:** Effect of *trans*-10, *cis*-12-CLA diet on milk production, milk lipid content, milk fatty acid profile, liver weight, and liver fatty acid profile.

Item	Treatment			*P* value
	Control	CLA	SEM	
Milk fat (weight %)	22.55	13.28	0.96	<0.01
Milk production (g)	3.51	2.12	0.53	NS^1^
Liver weight (g)	1.85	2.44	0.28	NS
Liver FAME^2^ (g)	0.26	0.47	0.03	<0.01
Milk fat (g/100 g FAME)				
<16:0	24.18	14.26	1.58	<0.01
16:0	22.42	17.99	0.62	<0.01
>16:0	50.48	66.68	1.67	<0.01
MUFA^3^	40.26	48.86	1.54	<0.01
* trans-*10,* cis-*12-CLA	ND^4^	1.42	0.05	NS
20:4n-6	0.54	1.32	0.09	<0.01
Liver (g/100 g FAME)				
16:0	12.88	21.51	0.68	<0.01
18:0	6.54	3.67	0.37	<0.01
18:1 *cis-*9	55.15	54.01	1.74	NS
*trans*-10, *cis*-12-CLA	ND	0.20	0.01	<0.01
18:3	1.51	1.13	0.07	<0.05
20:4n-6	5.11	1.91	0.42	<0.01

^1^Not significant.

^2^Fatty acid methyl esters.

^3^Monounsaturated fatty acids.

^4^Not detected.

**Table 3:** Top canonical pathways (FDR = 0.2, *P* < 0.001, and fold change >1.5) from IPA analysis of DEG in mammary tissue of lactating mice fed *trans*-10, *cis*-12-CLA.

Canonical pathways	−log (*P* value)	Downregulated^1^	Upregulated^1^
Acute-phase response signaling	1.88*E* + 00	81/178 (46%)	73/178 (41%)
Regulation of actin-based motility by rho	1.60*E* + 00	38/92 (41%)	39/92 (42%)
Glycosphingolipid biosynthesis-Ganglio series	1.54*E* + 00	10/58 (17%)	11/58 (19%)
RAR activation	1.51*E* + 00	78/187 (42%)	74/187 (40%)
LXR/RXR activation	1.44*E* + 00	32/86 (37%)	32/86 (37%)

^1^Fisher's exact test was used to calculate a *P* value determining the probability that the association between the genes in the dataset and the canonical pathway is explained by chance alone.

**Table 4:** Fold change in expression among the most up- and downregulated DEG in mammary tissue of lactating mice fed *trans*-10, *cis*-12-CLA.

Symbol	Name	CLA versus control	*P* value
Upregulated genes			

*Defb *	Defensin, beta1	4.6	0.004
*Hprt1 *	Hypoxanthine phosphoribosyl transferase 1	3.7	0.065
*Orm1 *	Orosomucoid 1	3.7	0.001
*Sphk1 *	Sphingosine kinase	3.6	0.011
*Kif26b *	Kinesin family member 26b	3.6	0.002
*Cp *	Ceruloplasmin (ferroxidase)	2.9	0.003
*Clca1 *	Chloride channel calcium activated 1	2.7	0.002
*Saa2 *	Serum amyloid A2	2.6	0.026

Downregulated genes			

*Mup4 *	Major urinary protein 4	−8.4	0.001
*Mup2 *	Major urinary protein 2	−4.6	0.001
*Ca3 *	Carbonic anhydrase III	−3.7	0.006
*Adipoq *	Adiponectin	−3.6	0.001
*Tbp *	TATA box binding protein	−3.2	0.011
*Scd *	Stearoyl-CoA desaturase	−3.1	0.001
*Elovl5 *	ELOVL family member 5, elongation of long chain fatty acids (yeast)	−2.7	0.001
*Smpd3 *	Sphingomyelin phosphodiesterase 3	−2.4	0.001
*Cfd *	Complement factor D (adipsin)	−2.4	0.001
*Tubg2 *	Tubulin, gamma 2	−2.2	0.001

**Table 5:** Verification of selected genes in mammary tissue with RT-qPCR.

Gene	Description	CLA versus control		
		Microarray	RT-qPCR	*P* value^1^
*Bax *	BCL2-associated X protein	1.11	−1.08	>0.10
*Stat5b *	Signal transducer and activator of transcription 5B	−1.39	−2.00	<0.01
*Rela *	v-rel reticuloendotheliosis viral oncogene homolog A	−1.51	−1.10	0.50
*Kras *	v-Ki-ras2 Kirsten rat sarcoma viral oncogene homolog	−1.28	−1.24	0.05
*B2m *	Beta-2 microglobulin	2.61	−1.57	<0.01
*Saa2 *	Serum amyloid A 2	2.58	5.47	<0.01
*Orm1 *	Orosomucoid 1	3.67	2.66	<0.01
*Cp *	Ceruloplasmin	2.94	1.09	>0.10
*Insig1 *	Insulin induced gene 1	−1.25	−1.11	>0.10
*Defb1 *	Defensin beta 1	4.56	2.31	<0.01
*C4b *	Complement component 4B (Childo blood group)	2.09	2.64	<0.01
*Ppar*γ**	Peroxisome proliferator activated receptor gamma	−1.34	−2.77	<0.01
*Mup2 *	Major urinary protein 2	−4.58	−11.64	<0.01
*Tnf *	Tumor necrosis factor	1.24	−1.50	0.09
*Saa1 *	Serum amyloid A 1	2.35	3.84	<0.01
*Orm2 *	Orosomucoid 2	2.28	3.81	<0.01
*Srebf1 *	Sterol regulatory element binding factor 1	−1.40	−1.23	0.01
*Scap *	SREBF chaperone	1.07	−1.05	>0.10
*Gpam *	Glycerol-3-phosphate acyltransferase, mitochondrial	−1.08	−1.39	0.01
*Scd *	Stearoyl-coenzyme A desaturase 1	−3.11	−3.78	<0.01
*Fasn *	Fatty acid synthase	−2.11	−2.31	<0.01

^1^RT-qPCR data.

**Table 6:** Top terms associated with the “mouse phenotype” and “disease” functions in the liver of mice fed *trans*-10, *cis*-12-CLA using the ToppGene suite application.

ID	Name	Source	*P* value	Term in query	Term in genome
(A) Upregulated genes					

*Mouse phenotype *					
(1) MP:0000388	Absent hair follicle inner root sheath		3.96*E* − 04	2	3
(2) MP:0008114	Abnormal Kupffer cell morphology		6.19*E* − 04	3	15
(3) MP:0008989	Abnormal liver sinusoid Morphology		7.45*E* − 04	4	36
(4) MP:0008248	Abnormal mononuclear phagocyte morphology		9.11*E* − 04	12	365
(5) MP:0002628	Hepatic steatosis		1.12*E* − 03	7	140
*Disease *					
(1) D015211	Zellweger syndrome	CTD	8.67*E* − 06	4	12
(2) 214100	Zellweger syndrome; ZS	OMIM	2.53*E* − 04	3	11
(3) 256000	Leigh syndrome; LS	OMIM	5.45*E* − 04	3	14
(4) D007888	Leigh Disease	CTD	6.75*E* − 04	3	15
(5) 20090112:Sabatti	Other metabolic traits	GWAS	2.15*E* − 03	3	22

(B) Downregulated genes					

*Mouse phenotype *					
(1) MP:0005328	Abnormal circulating creatinine level		5.30*E* − 05	9	71
(2) MP:0010011	Ectopic hippocampus pyramidal cells		1.41*E* − 04	3	5
(3) MP:0005311	Abnormal circulating amino acid level		3.29*E* − 04	11	130
(4) MP:0003205	Testicular atrophy		3.35*E* − 04	6	39
(5) MP:0009201	External male genitalia atrophy		6.01*E* − 04	2	2
*Disease *					
(1) 607850	Osteoarthritis susceptibility 3; OS3	OMIM	4.94*E* − 04	2	2
(2) 20090621:Hirschfield	Primary biliary cirrhosis	GWAS	6.99*E* − 03	2	6
(3) D008106	Liver cirrhosis, experimental	CTD	8.05*E* − 03	6	79
(4) C535531	Intervertebral disc disease	CTD	9.64*E* − 03	2	7
(5) 603932	Intervertebral disc disease; IDD	OMIM	9.64*E* − 03	2	7

**Table 7:** Top pathways affected in the liver of mice fed *trans*-10, *cis*-12-CLA determined by gene enrichment analysis using the ToppGene suite application.

ID	Name	Source	*P* value	Term in Query	Term in Genome
(A) Upregulated genes					

(1) Reactome electron transport chain	Genes involved in electron transport chain	MSigDB: C2.cp-Reactome	8.78*E* − 05	7	75
(2) Reactome glucose regulation of insulin secretion	Genes involved in Glucose Regulation of insulin secretion	MSigDB: C2.cp-Reactome	9.15*E* − 05	10	161
(3) PWY-561	Glyoxylate cycle II	BioCyc	1.58*E* − 04	4	20
(4) hsa01040	Biosynthesis of unsaturated fatty acids	KEGG pathway	2.34*E* − 04	4	22
(5) Reactome host interactions of HIV factors	Genes involved in host interactions of HIV factors	MSigDB: C2.cp-Reactome	2.94*E* − 04	8	120

(B) Downregulated genes					

(1) PW:0000028	Alanine and aspartate metabolic	Pathway ontology	1.43*E* − 03	3	10
(2) hsa00643	Styrene degradation	KEGG pathway	1.68*E* − 03	2	3
(3) BioCarta eryth pathway	Erythrocyte differentiation Pathway	MSigDB: C2.cp-BioCarta	4.95*E* − 03	3	15
(4) BioCarta stem pathway	Regulation of hematopoiesis by cytokines	MSigDB: C2.cp-BioCarta	4.95*E* − 03	3	15
(5) TYRFUMCAT pwy	Tyrosine degradation	BioCyc	5.42*E* − 03	2	5

**Table 8:** Top terms associated with GO functions in the liver of mice fed *trans*-10, *cis*-12-CLA using the ToppGene suite application.

GO category		*P* value	Term in query	Term in genome
(A) Upregulated genes				

*Molecular function *				
(1) GO:0016491	Oxidoreductase activity	3.41*E* − 07	23	727
(2) GO:0051087	Chaperone binding	1.85*E* − 06	6	40
(3) GO:0051082	Unfolded protein binding	3.33*E* − 05	8	131
(4) GO:0016462	Pyrophosphatase activity	5.26*E* − 04	18	798
(5) GO:0016818	Hydrolase activity, acting on acid anhydrides, in phosphorus-containing anhydrides	5.49*E* − 04	18	801
*Biological process *				
(1) GO:0043436	Oxoacid metabolic process	9.17*E* − 09	28	862
(2) GO:0019752	Carboxylic acid metabolic process	9.17*E* − 09	28	862
(3) GO:0044248	Cellular catabolic process	1.20*E* − 08	38	1,492
(4) GO:0006082	Organic acid metabolic process	1.40*E* − 08	28	879
(5) GO:0042180	Cellular ketone metabolic process	1.40*E* − 08	28	879
*Cellular component *				
(1) GO:0005739	Mitochondrion	3.03*E* − 13	45	1,482
(2) GO:0031966	Mitochondrial membrane	1.03*E* − 11	24	476
(3) GO:0005740	Mitochondrial envelope	2.64*E* − 11	24	498
(4) GO:0019866	Organelle inner membrane	2.79*E* − 10	20	379
(5) GO:0044429	Mitochondrial part	2.81*E* − 10	27	709

(B) Downregulated genes				

*Molecular function *				
(1) GO:0048037	Cofactor binding	2.69*E* − 05	18	277
(2) GO:0016823	Hydrolase activity, acting on acid carbon-carbon bonds, in ketonic substances	4.46*E* − 04	2	2
(3) GO:0016822	Hydrolase activity, acting on acid carbon-carbon bonds	4.46*E* − 04	2	2
(4) GO:0030170	Pyridoxal phosphate binding	1.15*E* − 03	6	56
(5) GO:0070279	Vitamin B6 binding	1.15*E* − 03	6	56
*Biological process *				
(1) GO:0032429	Regulation of phospholipase A2 activity	3.27*E* − 04	3	7
(2) GO:0007186	G-protein coupled receptor signaling pathway	6.04*E* − 04	38	1,008
(3) GO:0007156	Homophilic cell adhesion	9.83*E* − 04	10	141
(4) GO:0007205	Protein kinase C-activating G-protein coupled receptor signaling pathway	1.27*E* − 03	5	38
(5) GO:0009566	Fertilization	1.45*E* − 03	8	100
*Cellular component *				
(1) GO:0009986	Cell surface	1.94*E* − 04	23	472
(2) GO:0030136	Clathrin-coated vesicle	4.64*E* − 04	12	180
(3) GO:0045202	Synapse	5.01*E* − 04	22	473
(4) GO:0044456	Synapse part	9.17*E* − 04	17	338
(5) GO:0042734	Presynaptic membrane	1.15*E* − 03	6	56
