# Supplementary material for: Trans-10, cis 12-Conjugated Linoleic Acid-Induced Milk Fat Depression Is Associated with Inhibition of PPARγ Signaling and Inflammation in Murine Mammary Tissue
Source: J Lipids. 2013 May 14;2013:890343. doi: 10.1155/2013/890343 (PMC3666273; doi:10.1155/2013/890343)
Supplement: Supplementary file 1 — Supplementary Table 1: Top molecular and cellular functions (FDR=0.2, P<0.001 and fold change >1.5) up-regulated in the mammary tissue of lactating mice fed trans-10, cis-12-CLA. Supplementary Table 2: List of genes with stable gene expression across all the samples. Supplementary Table 3: Gene list for selecting internal controls without any interaction by IPA analysis. [file 890343.f1.doc]

### SUPPLEMENTAL MATERIALS

### *RNA Extraction*

Total RNA was extracted from 0.5 g tissue using ice-cold TRIZOL reagent (Invitrogen, Carlsbad, CA). The RNA concentrations were quantified with a NanoDrop ND-1000 spectrophotometer (NanoDrop Technologies, Wilmington, DE) and the quality was evaluated using a 2100 Bioanalyzer (Agilent Technologies, Santa Clara, CA, USA). Only samples with an RNA integrity number >8.0 were used for further analysis. Genomic DNA was removed with DNase using RNeasy Mini Kit columns (Qiagen, Valencia, CA). A portion of the assessed RNA was diluted to 100 mg/L using DNase-RNase free water prior to reverse transcription.

***Microarray analysis***

The MEEBO (Mouse Exonic Evidence based Oligonucleotide, Illumina Inc.) platform containing 38,467 single-spotted oligonucleotides targeting 35,302 genes was used for transcript profiling. Methods for amino-allyl labeling of cDNA, microarray hybridizations, and scanning were as described earlier (1). Hybridizations were performed in a dye-swap reference design. The reference was made by pooling RNA from all the samples. The cDNA was obtained by reverse transcriptase in a 30 μL reaction adding 10 μg RNA, 2 µL of random hexamer primers (3 μg/μL; Invitrogen Corp., CA), 1 µg oligo dT18 (Operon Biotechnologies, Huntsville, AL), and DNase-RNase-free water to a volume of 17.78 μL. The mixture was incubated at 65°C for 5 min and kept on ice for 3 min. To the mixture were added 12.2 μL solution composed of 6 μL 5X First-Strand Buffer, 3 μL 0.1 M DTT, 0.6 μL 100 mM dNTP mix (Invitrogen Corp.), 0.12 μL of 50 mM 5-(3-aminoallyl)-dUTP (Ambion, CA), 2 μL (100 U) of SuperScriptTM III RT (Invitrogen Corp.), and 0.5 μL of RNase Inhibitor (Promega, Agora, WI). The reaction was performed at 23°C for 1 min and 46°C for 9 h. The cDNA obtained was then treated with 10 µL 1 M NaOH, and incubated for 15 min at 65°C to remove residual RNA. The solution was neutralized by adding 10 µL 1 M HCl. The unincorporated 5-(3-aminoallyl)-dUTP and free amines were removed using a Qiagen PCR Purification Kit (Qiagen). Clean cDNA was vacuum-dried and resuspended in 4.5 µL 0.1 M Na2CO3 buffer (pH 9.0) and 4.5 µL of Amersham CyDye™ fluorescent dyes diluted in 60 µL of DMSO (Cy3 or Cy5; GE Healthcare, Waukesha, WI, USA). Binding of Cy dyes with 5-(3-aminoallyl)-dUTP incorporated into cDNA was obtained by incubation at room temperature for 1 h. The unbound dyes were removed using a Qiagen PCR Purification Kit (Qiagen) and clean labeled cDNA was measured by means of a NanoDrop ND-1000 spectrophotometer. Sample and reference were then vacuum-dried in the dark.

***Microarray hybridization and image acquisition***

Prior to hybridization, slides were re-hydrated, placed in an UV cross-linker, washed with 0.2% SDS solution, thoroughly rinsed with purified water to remove un-bound oligonucleotide, and pre-hybridized using a solution containing 1% albumin, 5 × SCC, and 0.1% SDS at 42 C° for ≥45 min with the aim of decreasing background. After pre-hybridization, slides were rinsed with abundant purified water and immersed in isopropanol for ~10s and spin-dried. Dried slides were immediately hybridized in a dye-swap-reference design (i.e., each sample was labeled twice with each of the two dyes and hybridized in each slide with the reference labeled with the opposite dye). Labeled cDNA of the sample was re-hydrated with 80 µL of hybridization buffer #1 (Ambion, Austin, TX) and mixed thoroughly. This solution was used to re-suspend the reference sample labeled with the opposite dye and mixed thoroughly in order to obtain a homogenous solution of the two labeled cDNA. Before hybridization, the labeled cDNA resuspension of the sample + reference was incubated at 90-95ºC for ca. 3 min to denature cDNA in order to increase the efficiency of oligo’s binding onto the slide.

Hybridizations were carried out using humidified slide chambers (Corning, Lowell, MA) with cover slips (LifterSlip; Thermo Scientific, Billerica, MA) at 42°C for ca. 40 hours in the dark. After hybridization, slides were removed from the chamber and washed for 5 min by agitation 3 times with wash buffers in the following order: 1×SSC and 0.2% SDS solution preheated at 42°C, 0.1×SSC and 0.2% SDS solution, and 0.1×SSC solution. Lastly, slides were inserted into a 50 mL tube, spin-dried and gassed with Argon to preserve dye from bleaching. Arrays were scanned with a ScanArray 4000 (GSI-Lumonics, Billerica, MA) dual-laser confocal scanner and images were processed and edited using GenePix 6.0 (Axon Instruments). Array quality was assessed using an in-house parser written in Perl language as previously described (2). Spots that received a -100 flag by GenePix 6.0 were removed from further analysis and background intensity was subtracted from the foreground intensity. Spots on the slide were considered “good” if the median intensity was ≥3×standard deviation above median background for each channel (i.e., dye). Spots were flagged “present” when both dyes passed the criteria, “marginal” if only one dye passed the criteria, or “absent” when both dyes failed to pass the criteria. Statistical analysis was conducted on oligos that were flagged as “present” and “marginal”.

### *RT-qPCR*

Sufficient cDNA was prepared to run all genes selected for verification of microarray results*.* Each cDNA was synthesized by RT-PCR and cDNA used for qPCR as previously described (3). Briefly, each cDNA was synthesized by RT using 100 ng RNA, 1 μL dT18 (Operon Biotechnologies, AL), 1 μL 10 mM dNTP mix (Invitrogen Corp.), 1 μL Random Primers (Invitrogen Corp., CA), and 7 μL DNase/RNase free water. The mixture was incubated at 65°C for 5 min and kept on ice for 3 min. A total of 9 μL of master mix composed of 4.5 μL 5X first-strand buffer, 1 μL 0.1 M DDT, 0.25 μL (100 U) of SuperScriptTM III RT (Invitrogen Corp.), and 0.25 μL of RNase Inhibitor (Promega, WI) and 3 mL DNase/RNase free water were added. The reaction was performed in an Eppendorf Mastercycler® Gradient using the following temperature program: 25°C for 5 min, 50°C for 60 min and 70°C for 15 min. The cDNA was then diluted 1:3 with DNase/RNase free water.

For qPCR analysis, 4 μL of diluted cDNA were combined with 6 μL of a mixture composed of 5 μL 1x SYBR Green master mix (Applied Biosystems, Foster City, CA), 0.4 μL each of 10μM forward and reverse primers, and 0.2 μL DNase/RNase free water in a MicroAmp™ Optical 384-Well Reaction Plate (Applied Biosystems). Each sample was run in triplicate and a 4-point relative standard curve (4-fold dilution) plus the non-template control were used (User Bulletin #2, Applied Biosystems). The reactions were performed in an ABI Prism 7900 HT SDS instrument (Applied Biosystems) using the following conditions: 2 min at 50 °C, 10 min at 95°C, 40 cycles of 15 s at 95°C, and 1 min at 60°C. The presence of a single PCR product was verified by the dissociation protocol using incremental temperatures to 95°C for 15 sec plus 65°C for 15 sec. Complete details regarding qPCR protocol can be found at <http://docs.appliedbiosystems.com/pebiodocs/04364014.pdf>. Data were analyzed with the 7900 HT Sequence Detection Systems Software (version 2.2.3, Applied Biosystems).

***Primer Design and Testing***

Primer Express 3.0 software (Applied Biosystems), optimized for use with Applied Biosystems qPCR Systems, was used for primers design using default features, except for the amplicon length, which was fixed at minimum of 100 bp . Primers were designed across exon junctions when possible to avoid amplification of genomic DNA. The exon junctions were uncovered blasting the sequence against murine genome (Genome Browser Gateway, 2008). Primers were aligned against publicly available sequences in NCBI (National Center for Biotechnology Information, 2008) and UCSC (Genome Browser Gateway, 2008). Prior to qPCR, primers were tested using the same protocol as for qPCR without the dissociation step in a 20µL reaction. Part of the PCR product was analyzed in a 2% agarose gel stained with ethidium bromide to assess presence of the product to an expected size and the presence of primer-dimer, with the remainder being purified using Qiaquick PCR purification kit (Qiagen) and sequenced at the Core DNA Sequencing Facility of the Roy J. Carver Biotechnology Center at the University of Illinois, Urbana. Only primers with high specificity evaluated by a single band on agarose gel, absence of primer-dimer, amplification of the target cDNA verified by sequencing, and a unique peak in the dissociation curve after qPCR reaction were used.

***Selection of Internal Control genes for qPCR***

The methodology for selection of internal control genes was as described earlier (3). Briefly, 31 genes showed stable expression across all the samples when tested with Genespring (Supplemental Table 3).Among these 26 genes were mapped by Ingenuity Pathway Analysis (IPA) and 14 genes did not have any interaction with each other (Supplemental Tables 3 and 4). geNorm software was then used to select 3 internal control genes based on stability analysis (4). The geometric mean of syntaxin 8 (*Stx8*), procollagen-lysine (*Plod3*) and neuroglobin (*Ngb*) was used to normalize the RT-qPCR data.

**REFERENCES**

1. Bionaz M, Periasamy K, Rodriguez-Zas SL, Everts RE, Lewin HA, Hurley WL, Loor JJ. Old and new stories: Revelations from functional analysis of the bovine mammary transcriptome during the lactation cycle. *PLoS One*. 7(3):1-14, 2012.
2. Loor JJ, Everts RE, Bionaz M, Dann HM, Morin DE, Oliveira R, Rodrigyez-Zas SL, Drackley JK, Lewin HA. Nutrition-induced ketosis alters metabolic and signaling gene networks in liver of periparturient dairy cows. *Physiol Genomics* 32: 105-116, 2007.
3. Bionaz M and Loor JJ. Identification of reference genes for quantitative real-time PCR in the bovine mammary gland during the lactation cycle. *Physiol Genomics* 29: 312-319, 2007.
4. Vandesompele J, De PK, Pattyn F, Poppe B, Van RN, De PA and Speleman F. Accurate normalization of real-time quantitative RT-PCR data by geometric averaging of multiple internal control genes. *Genome Biol* 3: RESEARCH0034, 2002.

**Supplemental Table 1.** Top molecular and cellular functions (FDR=0.2, P<0.001 and fold change >1.5) up-regulated in the mammary tissue of lactating mice fed *trans*-10, *cis*-12-CLA.

| **Category** | **No. of genes** | **P-values^1^** | | **Molecules** | |
| --- | --- | --- | --- | --- | --- |
| Lipid Metabolism | 11 | | 1.03E-03-4.82E-02 | | *TRPM4,B3GALT4,SEPP1,CFD,HNF1B,SCD,PCSK9,CHKA,ADIPOQ,FABP4,NAMPT* |
| Molecular Transport | 17 | | 1.03E-03-4.82E-02 | | *TRPM4,SCD,RELA,CFD,null,ADIPOQ,SLC1A3,STX1A,ODC1,HSPA8,GABRG2,SLC2A2,CHKA,FABP4,CLIC3,NAMPT,SMPD3* |
| Small Molecule Biochemistry | 23 | | 1.03E-03-4.82E-02 | | *TRPM4,CFD,SCD,RELA,ST3GAL2,SRR,PCSK9,GNAI1,PFN2,ADIPOQ,SLC1A3,CP,STX1A,ODC1,B3GALT4,SEPP1,HNF1B,SLC2A2,SMARCB1,CHKA,FABP4,NAMPT,SMPD3* |
| Cell Cycle | 12 | | 1.53E-03-4.82E-02 | | *RELA,PPP1CC,CCND2,SMC2,DNAJB4,SMARCB1,GNAI1,CHKA,LZTS2,CYR61,ATF2,AREG/AREGB* |
| Cell-To-Cell Signaling and Interaction | 13 | | 2E-03-4.82E-02 | | *RELA,SCD,RGS3,CTNNA3,ADIPOQ,L1CAM,SLIT2,NFASC,STX1A,ATF2,SMARCB1,CYR61,SYN1* |
| Cellular Assembly and Organization | 13 | | 2E-03-4.82E-02 | | *ZDHHC15,SCD,PFN2,CANX,L1CAM,SLIT2,NFASC,STX1A,SGCA,HNF1B,SMC2,CYR61,SYN1* |
| Cellular Movement | 11 | | 2E-03-4.6E-02 | | *PPP1CC,PDE2A,RGS3,RHOD,NR2F2,GNAI1,SLC1A3,LZTS2,L1CAM,NFASC,SLIT2* |
| Post-Translational Modification | 7 | | 4.27E-03-4.23E-02 | | *HSPA8,B3GALT4,SRR,ST3GAL2,PCSK9,HSP90AA1,CP* |
| Protein Folding | 2 | | 4.27E-03-4.27E-03 | | *HSPA8,HSP90AA1* |
| Gene Expression | 7 | | 4.82E-03-4.23E-02 | | *HNF1B,RELA,SMC2,NR2F2,IRF3,RARG,CARM1* |

^1^  The network genes associated with the biological functions in the Ingenuity Pathway Knowledge Base were considered for analysis by the IPA and the P-values were calculated based on Fisher’s exact test.

**Supplemental Table 2.** List of genes with stable gene expression across all the samples.

| **Gene ID** | **Symbol** | **Entrez Gene Name** | **Location** |
| --- | --- | --- | --- |
| NM_198018 | *Abr* | active BCR-related gene | Cytoplasm |
| NM_009709 | *Arnt* | aryl hydrocarbon receptor nuclear translocator | Nucleus |
| NM_007572 | *C1qa* | complement component 1, q subcomponent, A chain | Extracellular Space |
| NM_020263 | *Cacna2d2* | calcium channel, voltage-dependent, alpha 2/delta subunit 2 | Plasma Membrane |
| NM_080288 | *Elmo1* | engulfment and cell motility 1 | Cytoplasm |
| NM_010513 | *Igf1r* | insulin-like growth factor 1 receptor | Plasma Membrane |
| NM_025888 | *Kctd20* | potassium channel tetramerisation domain containing 20 | unknown |
| NM_175386 | *Lhfp* | lipoma HMGIC fusion partner | unknown |
| NM_028185 | *Lsm11* | LSM11, U7 small nuclear RNA associated | Nucleus |
| NM_011946 | *Map3k2* | mitogen-activated protein kinase kinase kinase 2 | Cytoplasm |
| NM_022414 | *Ngb* | neuroglobin | Cytoplasm |
| NM_145434 | *Nr1d1* | nuclear receptor subfamily 1, group D, member 1 | Nucleus |
| NM_030699 | *Ntng1* | netrin G1 | Extracellular Space |
| NM_025396 | *Pgls* | 6-phosphogluconolactonase | Cytoplasm |
| AY014830 | *Plod3* | procollagen-lysine, 2-oxoglutarate 5-dioxygenase 3 | Cytoplasm |
| NM_020032 | *Poll* | polymerase (DNA directed), lambda | Nucleus |
| NM_001003971 | *Senp7* | SUMO1/sentrin specific peptidase 7 | Nucleus |
| NM_183161 | *Slc17a9* | solute carrier family 17, member 9 | unknown |
| XM_125547 | *Slc22a16* | solute carrier family 22 (organic cation/carnitine transporter), member 16 | Plasma Membrane |
| NM_021537 | *Stk25* | serine/threonine kinase 25 | Cytoplasm |
| NM_018768 | *Stx8* | syntaxin 8 | Plasma Membrane |
| NM_029186 | *Tmem180* | transmembrane protein 180 | unknown |
| XM_486002 | *Tnrc6a* | trinucleotide repeat containing 6A | Nucleus |
| NM_009547 | *Zfp161* | zinc finger protein 161 homolog (mouse) | Nucleus |
| AK083392 | *Zfp866/Zfp869* | zinc finger protein 866 | unknown |
| NM_011747 | *Znf205* | zinc finger protein 205 | Nucleus |
| *D530014G21Rik** |  |  |  |
| *1810059H22Rik** |  |  |  |
| *2010013I23Rik** |  |  |  |
| *Myo1a** |  |  |  |
| *Slc22a16** | XM_125547 |  |  |

*Unmapped genes by Ingenuity Pathway Analysis.

**Suppl. Table 3.** Gene list for selecting internal controls without any interaction by IPA analysis.

| **Symbol** | **Entrez Gene Name** | **Location** | **Type(s)** |
| --- | --- | --- | --- |
| *Abr* | active BCR-related gene | Cytoplasm | other |
| *Cacna2d2* | calcium channel, voltage-dependent, alpha 2/delta subunit 2 | Plasma Membrane | ion channel |
| *Elmo1* | engulfment and cell motility 1 | Cytoplasm | other |
| *Lhfp* | lipoma HMGIC fusion partner | unknown | other |
| *Lsm11* | LSM11, U7 small nuclear RNA associated | Nucleus | other |
| *Ngb* | Neuroglobin | Cytoplasm | transporter |
| *Ntng1* | netrin G1 | Extracellular Space | other |
| *Plod3* | procollagen-lysine, 2-oxoglutarate 5-dioxygenase 3 | Cytoplasm | enzyme |
| *Poll* | polymerase (DNA directed), lambda | Nucleus | enzyme |
| *Senp7* | SUMO1/sentrin specific peptidase 7 | Nucleus | peptidase |
| *Slc17a9* | solute carrier family 17, member 9 | unknown | other |
| *Stx8* | syntaxin 8 | Plasma Membrane | other |
| *Tmem180* | transmembrane protein 180 | unknown | other |
| *1200003I07RIK* | -- | unknown | other |
